# Minocycline-Induced Ocular Ochronosis

**DOI:** 10.7759/cureus.43307

**Published:** 2023-08-10

**Authors:** Carla Baaklini, Natasha Kesav, William Reinhart

**Affiliations:** 1 Ophthalmology, University of Missouri, Columbia, USA; 2 Ophthalmology, University Hospitals Cleveland Medical Center, Cleveland, USA

**Keywords:** retinal pigment epithelium deposits, scleral hyperpigmentation, hyperpigmentation, minocycline, ocular ochronosis

## Abstract

We report a case of minocycline-induced ocular ochronosis with scleral, retinal, and cutaneous manifestations. A 65-year-old male who had taken minocycline for four years to treat hidradenitis suppurativa, an inflammatory skin condition affecting the apocrine sweat glands and hair follicles, presented for evaluation of discoloration of bilateral sclera, nail beds, and gingiva. Ophthalmic evaluation revealed intact visual acuity, diffuse blue-gray hyperpigmentation of the sclera, more pronounced overlying insertions of the horizontal muscles, without any scleral thinning. Macular optical coherence tomography and fundus exam revealed a blue hue to the underlying choroid with dark deposits in the retinal pigment epithelium. Despite drug discontinuation, after six years the discoloration persisted. Management was directed towards patient tolerability.

## Introduction

Minocycline is a tetracycline antibiotic used to treat a variety of conditions such as hidradenitis suppurativa, chronic blepharitis, rheumatoid arthritis, acne vulgaris, and bacterial infections such as chlamydia, gonococcus, nocardia, and syphilis. One of its adverse effects includes hyperpigmentation of the skin and mucus membranes which has been described as both dose-independent [1] and dose-dependent [2]. Scleral hyperpigmentation can be permanent or may fade over time with discontinuation of the medication [3,4], without changes to visual acuity.

## Case presentation

A 65-year-old male presented for evaluation of blue-gray discoloration of both eyes. His medical history was significant for hidradenitis suppurativa treated indefinitely with minocycline, 100 mg twice daily, for a total duration of four years. The patient had an ocular history of dry eye syndrome and no history of inflammatory eye disorders.

**Figure 1:**
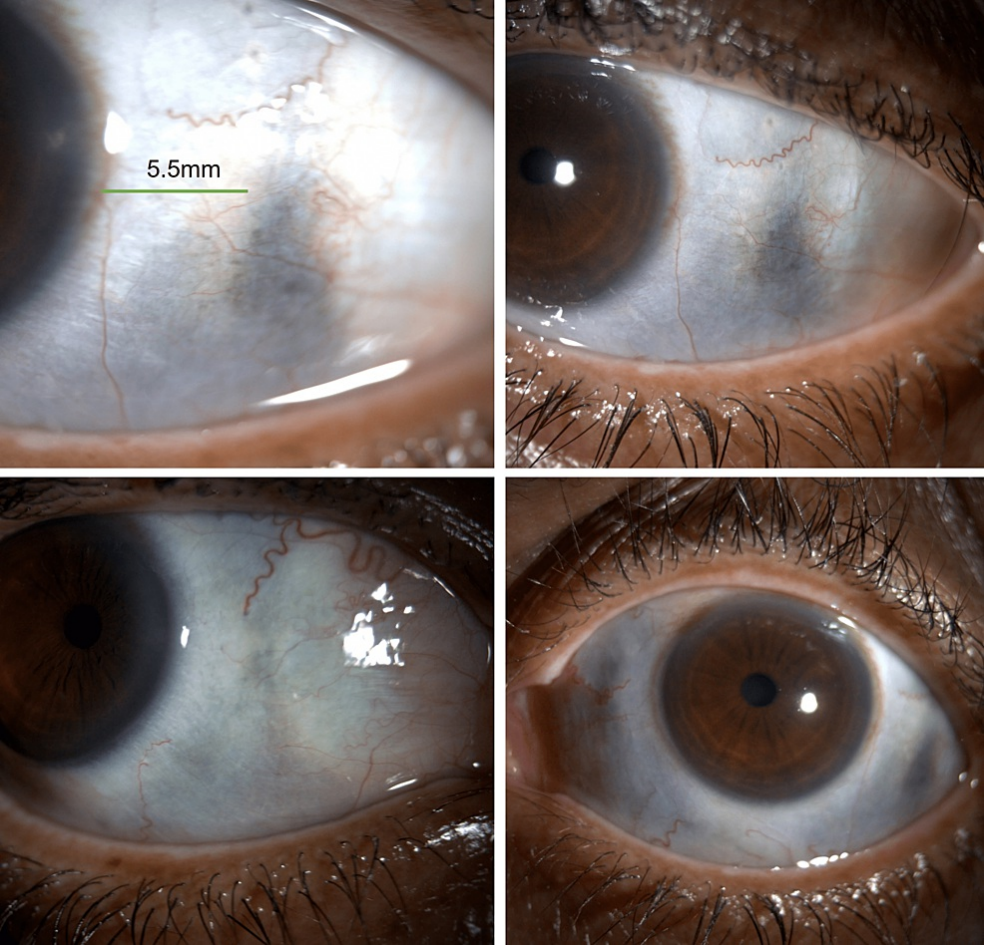
Blue discoloration of sclera with prominence in palpebral aperture. There is an area 5.5 mm posterior to the limbus where the scleral coloration becomes more prominent.

**Figure 2:**
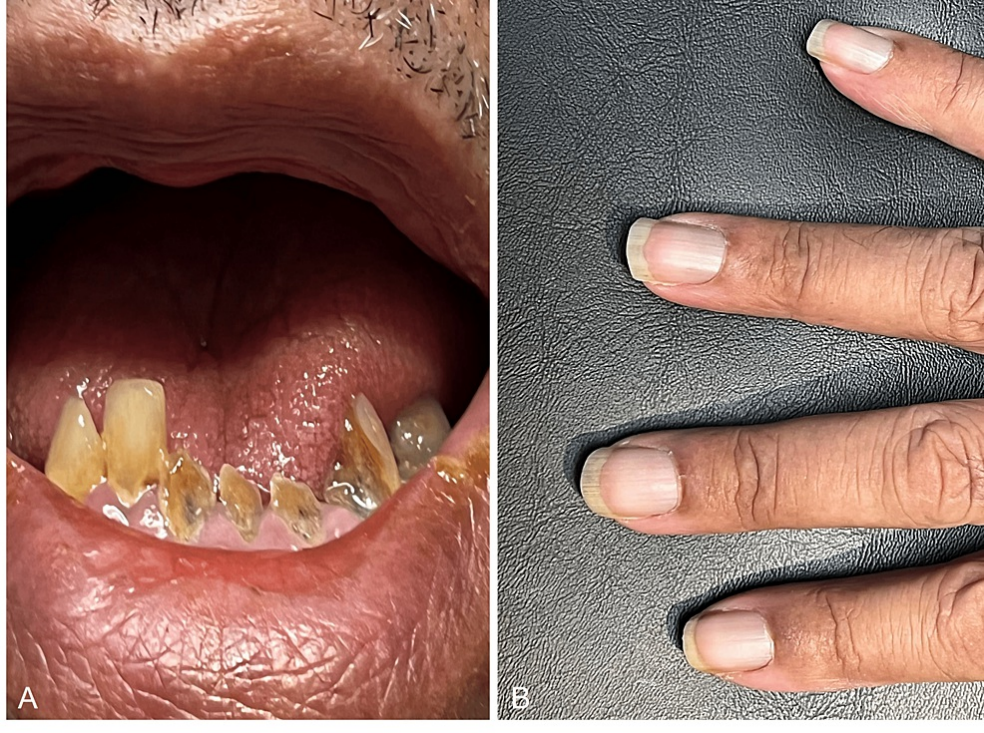
Blue discoloration of oral mucosa (A) and fingernails (B).

**Figure 3:**
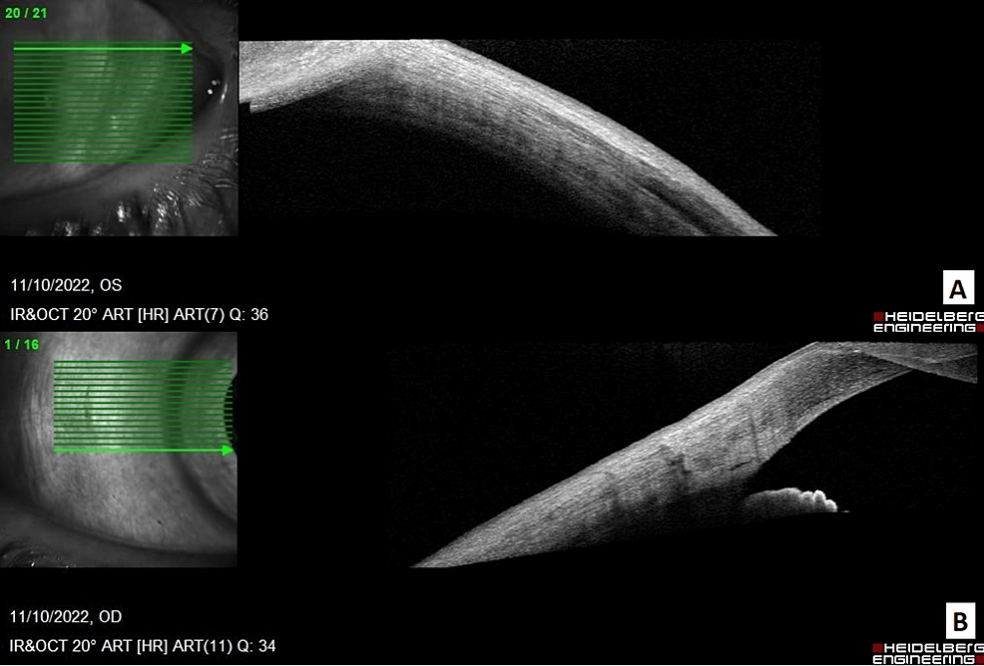
A: Anterior segment optical coherent tomography of the temporal left eye showing lack of scleral thinning. B: Anterior segment optical coherent tomography showing lack of scleral thinning nasally.

**Figure 4:**
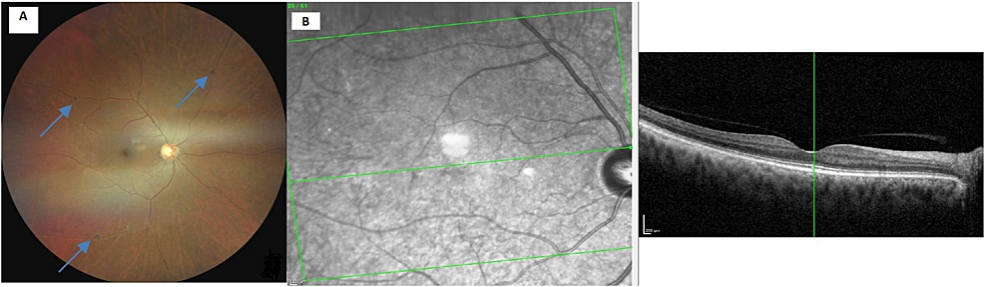
A: Right eye fundus photography showing bluish appearance to macula. B: Pigmented retinal pigment epithelium deposits seen on optical coherence tomography.

Visual acuity was 20/25 right eye (OD) and 20/40 left eye (OS). Intraocular pressure was 16 mm Hg OD and 17 mm Hg OS. Slit-lamp examination revealed 1+ nuclear sclerosis in both eyes. External examination revealed blue-gray discoloration of the nail beds, gingiva, and bilateral sclera (Figures 1, 2). There was diffuse, patchy discoloration of the sclera that was densest overlying the insertions of the horizontal muscles. Anterior segment optical coherent tomography showed a lack of scleral thinning (Figure 3). Macular optical coherence tomography and fundus exam revealed a blue hue to the macula with associated speckled dark deposits in the pigmented retinal pigment epithelium (Figure 4) in the absence of inflammatory signs of periphlebitis. These retinal findings were similar in both eyes but more distinct in the right eye. Due to the choroid not being noted to be hyperreflective, it was concluded that there was not a pigmented choroidal nevus. The patient had no documented ocular exams prior to starting minocycline, however his driver’s license photo taken six years prior to the start of minocycline did not demonstrate scleral pigmentation as described. Dermatologic exams prior to minocycline initiation did not document any discoloration of skin or fingernails. Given the unknown, long-term impact of minocycline-induced ocular hyperpigmentation, the patient was recommended to discontinue minocycline and begin methotrexate, 10 mg weekly, with folic acid to manage his hidradenitis suppurativa.

## Discussion

Minocycline-induced scleral pigmentation was first described by Fraunfelder et al. in 1997 [5] in seven eyes. These findings were in patients who also demonstrated hyperpigmentation in other areas of the body that included fingernails, bone, teeth, skin, thyroid, and/or mucosa [1,5]. Scleral pigmentation has been found to occur in 2.4% to 14.8% of users of this medication and more likely occurs at doses of at least 100 mg daily [3]. Minocycline’s lipophilicity is likely the causative factor predisposing to hyperpigmentation in the sclera as well as the conjunctiva and retina [6].

The pathophysiology of hyperpigmentation is not solidified, but it is thought to be related to a metabolite of minocycline that chelates with iron in macrophages [2] and/or thought to form a complex with melanin in the epidermis [7]. Hyperpigmentation has been divided into three main types [2,3]. Type I manifests as blue-pigmented scars, type II as blue-pigmented skin that most commonly involves the extremities, and type III as brown pigment of sun-exposed skin. Type I stains positive for iron, type II for iron and melanin, and type III for melanin [8]. Types II and III have been found to be dose-dependent, unlike type I [9].

This case report highlights the ocular side effects with scleral pigmentation and suspected retinal changes. The scleral hyperpigmentation classically starts at the limbus, is more prominent in the palpebral aperture, and is a blue-gray band that is 3 to 5 mm wide [5]. Conjunctival manifestations of minocycline have been described as brown or black cysts [10,11]. These effects have been noted as soon as a few weeks of being on the medication [8], although they more commonly occur after over one year in a dose-dependent manner [9]. Damage to retinal photoreceptors is thought to be a late manifestation of treatment.

Ocular manifestations are typically not isolated and occur with other areas of the body [12]. The effects of the medication on the skin and scleral surface can take up to four or more years to fade, however, it can be intensified with exposure to UV rays [3]. In some studies, the pigment has been permanently deposited [5]. No effect on visual acuity has been identified to date [3,4].

Multimodal imaging has been utilized to characterize these patterns. Scleral thickness, when evaluated with ocular coherence tomography has been noted to be normal [4,13]. The aforementioned conjunctival cysts have been identified via cobalt blue filter autofluorescence [10] and with yellow autofluorescence showing eosinophilic amorphous concretions [14]. Regarding retinal manifestations, a case of macula involvement described green wavelength fundus autofluorescence as having a hyperautofluorescence that was speckled in appearance. Ocular coherence tomography showed pigmented epithelial detachments [15]. Another case of retinal involvement did not note any hyper- or hypo-fluorescence on angiography and only noted nodular deposits in the retinal pigment epithelium [16].

Differential diagnoses

Systemic medical conditions may mimic minocycline-induced hyperpigmentation. The differential for blue-colored hyperpigmentation of the scleral surface includes collagen disorders, such as Marfan syndrome, osteogenesis imperfecta, and Ehlers-Danlos syndrome. These conditions can also include pathologies of the musculoskeletal, cardiovascular, nervous, and integumentary systems [17,18]. Alkaptonuria, an autosomal recessive condition, is caused by a mutation in a gene involved in the degradation pathway of the amino acid homogentisate, causing onchronosis, or blue-brown discoloration of urine, cartilage, and sclera [19]. Adrenal insufficiency, which is associated with hemodynamic instability, has also been found to cause hyperpigmentation which affects the skin and conjunctiva [20]. The patient in this case report had no such medical history or laboratory findings.

Other ocular conditions may present with a similar blue-gray pattern. This includes melanocytic disorders that can trigger this condition, i.e., oculodermal melanocytosis, also known as Nevus of Ota, which leads to gray or blue patches on the skin and epithelium. This condition can increase the risk of developing ocular melanoma and glaucoma. Ocular melanomas with scleral extension may look similar to medication-induced scleral hyperpigmentation. Necrotizing scleritis may also cause a blue-colored appearance, however unlike other conditions that can cause scleral discoloration, scleral thinning is present [19].

Management

Management is often directed towards patient tolerability. Cutaneous hyperpigmentation can be treated with picosecond or Q-switched lasers [8], but an intervention for scleral pigmentation has not been identified. Patients are routinely advised to discontinue the medication due to possible permanent change in the scleral color [7].

## Conclusions

This report highlights the ocular and cutaneous findings of minocycline-induced hyperpigmentation after four years of minocycline treatment in a 65-year-old male with hidradenitis suppurativa. Ophthalmologists may be the first to note scleral discoloration. It is important to monitor for hyperpigmentation in patients taking minocycline, as the discoloration has been reported to be permanent with long-term use.
